# Co-crystallization of a neutral mol­ecule and its zwitterionic tautomer: structure and Hirshfeld surface analysis of 5-methyl-4-(5-methyl-1*H*-pyrazol-3-yl)-2-phenyl-2,3-di­hydro-1*H*-pyrazol-3-one 5-methyl-4-(5-methyl-1*H*-pyrazol-2-ium-3-yl)-3-oxo-2-phenyl-2,3-di­hydro-1*H*-pyrazol-1-ide monohydrate

**DOI:** 10.1107/S2056989019004389

**Published:** 2019-04-05

**Authors:** Abdullah M. Asiri, Khalid A. H. Alzahrani, Hassan M. Faidallah, Khalid A. Alamry, Mukesh M. Jotani, Edward R. T. Tiekink

**Affiliations:** a Center of Excellence for Advanced Materials Research, King Abdulaziz University, PO Box 80203, Jeddah 21589, Saudi Arabia; b Chemistry Department, Faculty of Science, King Abdulaziz University, PO Box 80203, Jeddah 21589, Saudi Arabia; c Department of Physics, Bhavan’s Sheth R. A. College of Science, Ahmedabad, Gujarat 380001, India; d Research Centre for Crystalline Materials, School of Science and Technology, Sunway University, 47500 Bandar Sunway, Selangor Darul Ehsan, Malaysia

**Keywords:** crystal structure, pyrazolone, pyrazole, tautomer, Hirshfeld surface analysis

## Abstract

The title compound, 2C_14_H_14_N_4_O·H_2_O, comprises a neutral mol­ecule containing a central pyrazol-3-one ring flanked by an N-bound phenyl group and a C-bound 5-methyl-1*H*-pyrazol-3-yl group (at positions adjacent to the carbonyl substituent), its zwitterionic tautomer, whereby the N-bound proton of the central ring is now resident on the pendant ring, and a water mol­ecule of crystallization. Besides systematic variations in geometric parameters, the two independent organic mol­ecules have broadly similar conformations, as seen in the dihedral angle between the five-membered rings [9.72 (9)° for the neutral mol­ecule and 3.32 (9)° for the zwitterionic tautomer] and in the dihedral angles between the central and pendant five-membered rings [28.19 (8) and 20.96 (8)° (neutral mol­ecule); 11.33 (9) and 11.81 (9)°]. In the crystal, pyrazolyl-N—H⋯O(carbon­yl) and pyrazolium-N—H⋯N(pyrazol­yl) hydrogen bonds between the independent organic mol­ecules give rise to non-symmetric nine-membered {⋯HNNH⋯NC_3_O} and {⋯HNN⋯HNC_3_O} synthons, which differ in the positions of the N-bound H atoms. These aggregates are connected into a supra­molecular layer in the *bc* plane by water-O—H⋯N(pyrazolide), water-O—H⋯O(carbon­yl) and pyrazolyl-N—H⋯O(water) hydrogen bonding. The layers are linked into a three-dimensional architecture by methyl-C—H⋯π(phen­yl) inter­actions. The different inter­actions, in particular the weaker contacts, formed by the organic mol­ecules are clearly evident in the calculated Hirshfeld surfaces, and the calculated electrostatic potentials differentiate the tautomers.

## Abstract 2

The title compound comprises a neutral mol­ecule, its zwitterionic tautomer whereby the N-bound proton of the central ring is now resident on the pendant ring, and a water mol­ecule of crystallization. Conventional hydrogen bonding leads to supra­molecular layers in the crystal.

## Chemical context   

Mol­ecules related to the title compound, *i.e*. containing a pyrazolone ring, are of particular inter­est owing to their pharmaceutical potential. Applications in this context include their possible utilization as cardiovascular drugs (Higashi *et al.*, 2006 ▸), as hypoglycemic agents (Das *et al.*, 2008 ▸) and as anti-inflammatory and analgesic agents (Badawey & El-Ashmawey, 1998 ▸). This class of compound has also been evaluated as anti-microbials (Sahu *et al.*, 2007 ▸) and display fungicidal activities (Singh & Singh, 1991 ▸). In the course of studies in this area, the title compound, which has been synthesized previously (Kumar *et al.*, 1995 ▸), was characterized crystallographically on a crystal isolated from an ethanol solution and found to contain neutral and zwitterionic tautomers.
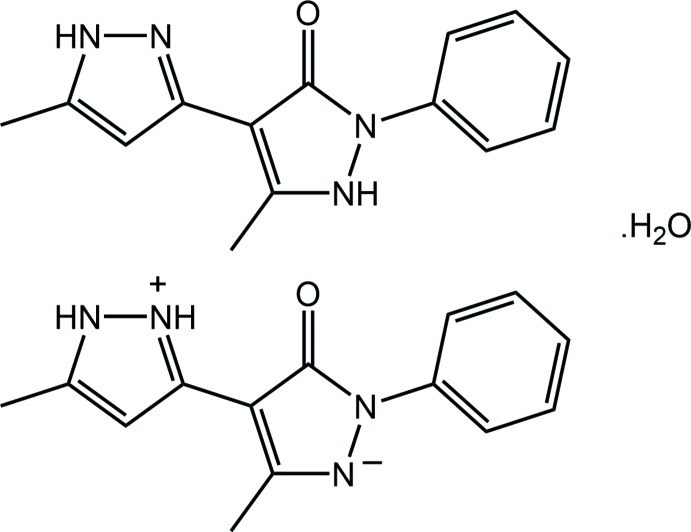


Tautomerism relates to a phenomenon whereby isomeric structures undergo inter-conversion by the migration of, typically, an atom, often a proton, or small group within the mol­ecule. While different tautomers can co-exist in solution, in crystals usually only one form is found (Rubčić *et al.*, 2012 ▸). Notable examples of tautomers crystallizing in the same crystal begin with biologically relevant isocytosine (Sharma & McConnell, 1965 ▸) and the histidine residue in the structure of l-His-Gly hemihydrate (Steiner & Koellner, 1997 ▸). Such behaviour has also been observed, for example, in a synthetic compound, namely, *N*-(3-hy­droxy­salicyl­idene)-4-meth­oxy­aniline (Pizzala *et al.*, 2000 ▸). Herein, the crystal and mol­ecular structures of the title compound, (I), are described along with an analysis of the calculated Hirshfeld surfaces.

## Structural commentary   

**Figure 1:**
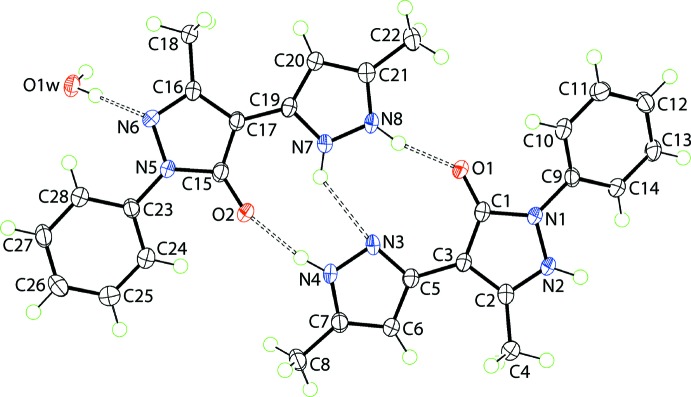
The mol­ecular structures of the constituents of (I), showing displacement ellipsoids at the 70% probability level. The pyrazolyl-N4, N8—H⋯O2, O1(carbon­yl), pyrazolium-N7—H⋯N3(pyrazol­yl) and water-O—H⋯N6(pyrazolium) hydrogen bonds are shown as dashed lines. Note the non-symmetric nine-membered {⋯HNNH⋯NC_3_O} and {⋯HNN⋯HNC_3_O} synthons formed between the neutral and tautomeric mol­ecules.

The crystal of (I), Fig. 1 ▸, comprises a neutral mol­ecule of 5-methyl-4-(5-methyl-1*H*-pyrazol-3-yl)-2-phenyl-2,3-di­hydro-1*H*-pyrazol-3-one, its zwitterionic tautomer 5-methyl-4-(5-methyl-1*H*-pyrazol-2-ium-3-yl)-3-oxo-2-phenyl-2,3-di­hydro-1*H*-pyrazol-1-ide and a mol­ecule of water. Evidence of tautomerism is twofold. Firstly, in the nature of the hydrogen-bonding inter­actions operating in the crystal; see *Supra­molecular features*. Secondly, in small but systematic variations in key geometric parameters, Table 1 ▸. Thus, the N5—N6 bond in the zwitterion is longer than the equivalent N1—N2 bond in the neutral species with a concomitant shortening of the C16—N6 and lengthening of the C16—C17 bonds with respect to the bonds in the neutral species. The most notable changes in angles relate to protonation/deprotonation. Thus, the angle at the protonated N2 atom is greater than the angle at the deprotonated N6, and the same is true for the angles subtended at the N7 and N3 atoms. Mol­ecules in (I) may also exhibit rotational isomerism about the C3—C5 and C17—C19 bonds. In the present case, the carbonyl group is *syn* with the pendent ring imine-N atom in the neutral mol­ecule and is designated the NH-*Z* form (Kumar *et al.*, 1995 ▸); the zwitterionic tautomer has a similar conformation.

**Figure 2:**
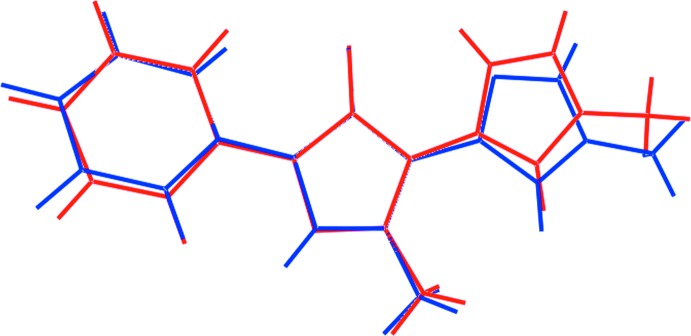
Overlay diagram of the two organic mol­ecules in (I): neutral mol­ecule (blue image) and zwitterion (red). The mol­ecules have been overlapped so that the five-membered rings are coincident.

The differences in geometric parameters characterizing the two independent organic mol­ecules in (I) notwithstanding, the mol­ecules present very similar conformations as highlighted in the overlay diagram, Fig. 2 ▸. Thus, the r.m.s. deviations of the fitted atoms of the N1, N3, N5 and N7 rings are 0.019, 0.003, 0.006 and 0.006, respectively. The dihedral angle between the N1 and N3 rings is 9.72 (9)°, and that between each of these and the appended phenyl ring are 28.19 (8) and 20.96 (8)°. The comparable values for the zwitterionic tautomer are 3.32 (9), 11.33 (9) and 11.81 (9)°, respectively, indicating that this mol­ecule is closer to planar, as vindicated by the difference in the N2—N1—C9—C14 and N6—N5—C23—C28 torsion angles of 26.2 (2) and −7.4 (2)°, respectively.

## Supra­molecular features   

**Figure 3:**
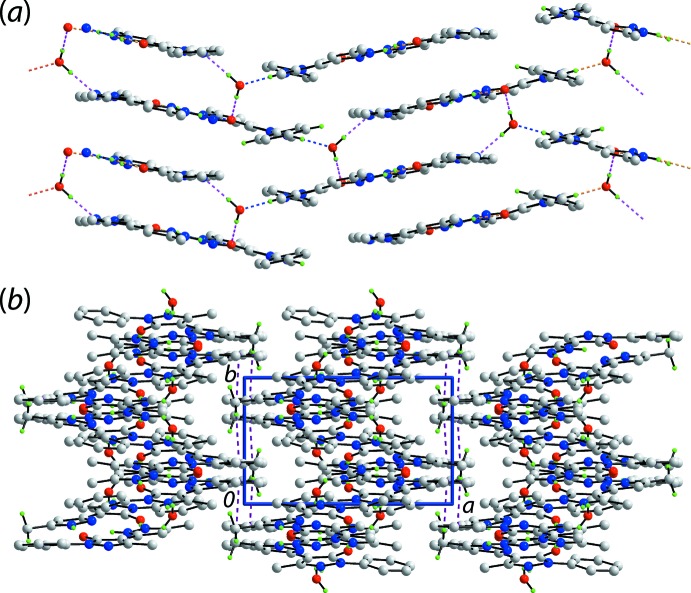
Supra­molecular association in the crystal of (I): (*a*) a view of the supra­molecular layer in the *bc* plane whereby the dimeric aggregates shown in Fig. 1 ▸ are connected by water-O1*W*⋯O1(carbon­yl), water-O1*W*⋯N6(pyrazolide) (pink dashed lines) and pyrazolyl-N2—H⋯O1*W*(water) hydrogen bonds (blue dashed lines) and (*b*) a view of the unit-cell contents shown in projection down the *c* axis. The C—H⋯π inter­actions are shown as purple dashed lines. In both images, the non-participating and non-acidic H atoms are omitted.

The mol­ecular packing of (I) features substantial conventional hydrogen-bonding inter­actions and the description of these can be conveniently divided by discussing inter­actions without the involvement of the water mol­ecule of crystallization and then considering the role of the water mol­ecule. Table 2 ▸ lists the geometric parameters of the specific inter­molecular inter­actions in the crystal of (I). There are three hydrogen bonds formed between the two organic mol­ecules comprising the asymmetric unit and these involve the three outer-ring amine-H atoms as donors and a ring-N and the two carbonyl-O atoms as acceptors. The resulting pyrazolyl-N4, N8—H⋯O1, O2(carbon­yl) and pyrazolium-N7—H⋯N3(pyrazol­yl) hydrogen bonds give rise to non-symmetric nine-membered {⋯HNNH⋯NC_3_O} and {⋯HNN⋯HNC_3_O} synthons, differing only in the relative placement of one of the ring-N-bound H atoms, Fig. 1 ▸. The two-mol­ecule aggregates are assembled into a supra­molecular layer in the *bc* plane by hydrogen bonding involving the water mol­ecule, which functions as a donor to the pyrazolide-N6 and carbonyl-O1 atoms and as an acceptor from a pyrazolyl-N2 atom, Fig. 3 ▸ *a*. Further consolidation of the layers is provided by π–π inter­actions with the shortest of these involving the N1-pyrazolyl and phen­yl(C9–C14)^i^ rings [inter-centroid separation = 3.5810 (9) Å, inter-planar angle = 6.29 (8)° for symmetry operation: (i) 1 − *x*, 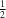 + *y*, 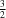 − *z*]. As shown in Fig. 3 ▸ *b*, the points of contact between layers involve methyl-C8—H⋯π inter­actions with the symmetry-related phenyl (C23–C28) ring, Table 2 ▸, resulting in a three-dimensional architecture.

## Hirshfeld surface analysis   

The Hirshfeld surface calculations for (I) were calculated employing *Crystal Explorer* (Turner *et al.*, 2017 ▸) and were conducted in accord with recent studies (Tan *et al.*, 2019 ▸) to investigate the influence of inter­molecular inter­actions between the neutral and zwitterionic tautomers, along with the water mol­ecule of crystallization, on the mol­ecular packing.

**Figure 4:**
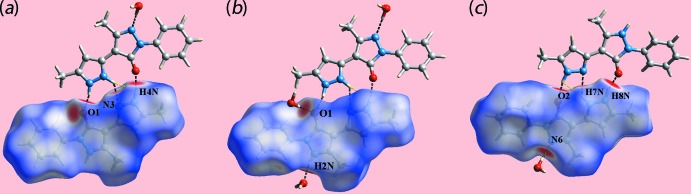
Views of the Hirshfeld surface for (I) mapped over *d*_norm_ in the range −0.527 to +1.288 arbitrary units for (*a*) and (*b*) the neutral tautomer and (*c*) in the range −0.527 to +1.367 arbitrary units for the zwitterion and a short intra-atomic H⋯H contact by a yellow dashed line.

**Figure 5:**
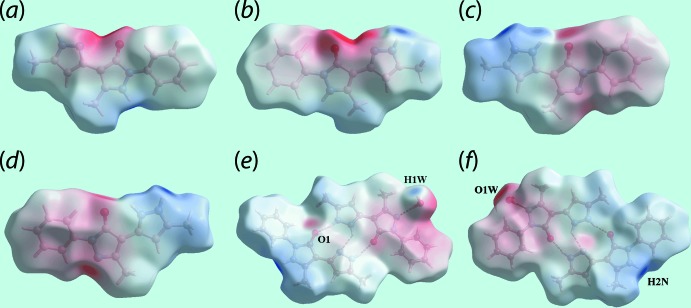
Views of Hirshfeld surface mapped over the calculated electrostatic potential for (*a*) and (*b*) the neutral tautomer in the range −0.157 to +0.225 atomic units (a.u.), (*c*) and (*d*) zwitterionic tautomer in the range −0.152 to +0.259 a.u., and (*e*) and (*f*) for the overall structure in the range −0.166 to +0.250 a.u.. The red and blue regions represent negative and positive electrostatic potentials, respectively.

The donors and acceptors of the hydrogen bonds summarized in Table 2 ▸ and discussed in the previous section are clearly evident as the broad and bright-red spots on the Hirshfeld surface mapped over *d*_norm_ for the neutral tautomer in Fig. 4 ▸ *a* and *b* and for the zwitterionic tautomer in Fig. 4 ▸ *c*. In addition to these, a short intra­molecular H⋯H contact between symmetry-related pyrazolyl-H4*N* and H7*N* atoms (Table 3 ▸) is viewed as faint-red spots near these atoms on the *d*_norm_-mapped Hirshfeld surfaces of the tautomers in Fig. 4 ▸ *a* and *c*. It is clear from the views of the Hirshfeld surfaces mapped over the calculated electrostatic potential for neutral mol­ecule in Fig. 5 ▸ *a* and *b*, and for the zwitterionic tautomer in Fig. 5 ▸ *c* and *d*, that they have quite distinct charge distributions on their surfaces. The presence of non-protonated pyrazolyl-N3 and N6 atoms results in a pronounced electronegative regions adjacent to carbonyl-O1 atom in the neutral form and opposite to carbonyl-O2 atom in the zwitterion as shown by the intense-red regions in Fig. 5 ▸ *a*–*d*; hence facilitating the charge-assisted hydrogen bonds with pyrazolyl-N7 and water-O1*W* atoms, respectively. The donors and acceptors of inter­molecular water-O1*W–*-H⋯O1(carbon­yl) and pyrazolyl-N2—H⋯O1*W*(water) are also viewed as blue and red regions in Fig. 5 ▸ *e* and *f*.

**Figure 6:**
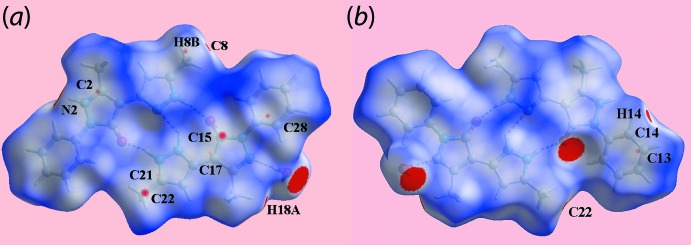
Views of Hirshfeld surfaces mapped over *d*_norm_ for the overall structure in the range −0.031 to +1.343 arbitrary units.

**Figure 7:**
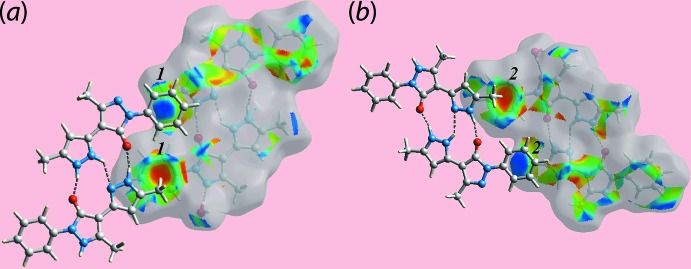
Views of Hirshfeld surfaces mapped with the shape-index property highlighting the donors (labelled ‘1′) and acceptors (‘2′) of C—H⋯π contacts through blue and red regions, respectively.

The short inter­atomic contacts characterizing weak inter­molecular inter­actions in the crystal of (I) are viewed as characteristic red spots near the involved atoms on the Hirshfeld surface of the overall structure by modifying (making more sensitive) the *d*_norm_ range, see Fig. 6 ▸ and Table 3 ▸. The short intra- and inter-layer C⋯C contacts formed by the methyl-C22 atom with methyl-C8 and pyrazolyl-C15 atoms (Table 3 ▸) are viewed as small red spots near these atoms in Fig. 6 ▸ *a*. The presence of faint-red spots near pyrazole-N2, C2, C17, C21 and phenyl-C13, C14 atoms in Fig. 6 ▸ represent their participation in short inter­atomic C⋯C and C⋯N contacts (Table 3 ▸) arising from π–π contacts between the N1-pyrazolyl and phenyl (C9–C14), and pyrazolyl-N5 and pyrazolyl-N7 rings (Table 5 ▸). In addition to this, the influence of short inter­atomic H⋯H and C⋯H/H⋯C contacts (Table 3 ▸) on the packing is also evident as the faint-red spots near methyl-H8*B* and phenyl-C28 atoms, and the spots near the methyl-H18*A* and phenyl-C14, H14 atoms in Fig. 6 ▸. The involvement of the methyl-H8*A* and H8*B* atoms as the donors and the phenyl (C23–C28) ring as the acceptor in the C—H⋯π contacts are also confirmed from the Hirshfeld surface mapped with the shape-index property through blue and red regions, respectively, in Fig. 7 ▸.

**Figure 8:**
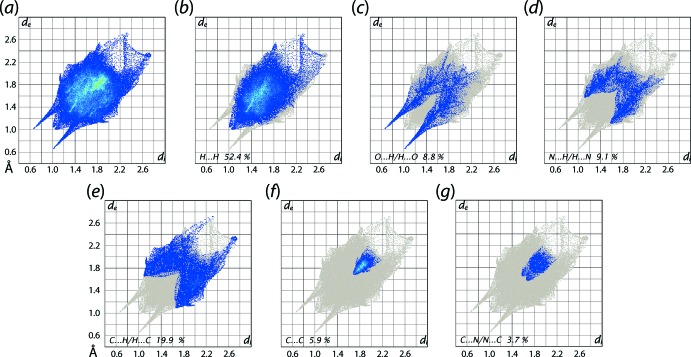
(*a*) The full two-dimensional fingerprint plot for (I) and (*b*)–(*f*) those delineated into H⋯H, O⋯H/H⋯O, N⋯H/H⋯N, C⋯H/H⋯C, C⋯C and C⋯N/N⋯C contacts, respectively.

The overall two dimensional fingerprint plot in Fig. 8 ▸ *a* and those delineated into H⋯H, O⋯H/H⋯O, N⋯H/H⋯N and C⋯H/H⋯C, C⋯C and C⋯N/N⋯C contacts for the overall structure are illustrated in Fig. 8 ▸ *b*–*g* and the percentage contributions from the different inter­atomic contacts to the Hirshfeld surfaces of the neutral tautomer, zwitterion and the overall structure are summarized in Table 4 ▸.

In the fingerprint plot delineated into H⋯H contacts, Fig. 8 ▸ *b*, the short contact involving the methyl-H18*A* and phenyl-H14 atoms, Table 3 ▸, are viewed as the pair of two adjacent short peaks at *d*_e_ + *d*_i_ ∼2.1 Å while the points corresponding to the H13⋯H18*A* contact are merged within the plot. The involvement of the water mol­ecule in the N—H⋯O hydrogen bond results in a pair of long spikes at *d*_e_ + *d*_i_ ∼1.8 Å in the fingerprint plot delineated into O⋯H/H⋯O contacts, Fig. 8 ▸ *c*; these encompass the pair of spikes corresponding to the O—H⋯O hydrogen bond involving carbonyl-O1 atom. The percentage contribution from these contacts to the Hirshfeld surface of the overall structure is less than the individual tautomers (Table 4 ▸) as the atoms of the organic components comprising the asymmetric unit are self-associated by hydrogen bonds as well as participating in hydrogen bonding with the water mol­ecule.

The fingerprint plot delineated into N⋯H/H⋯N contacts in Fig. 8 ▸ *d* shows inter­atomic distances are at van der Waals separations or longer in the crystal. The significant 19.9% contribution from C⋯H/H⋯C contacts to the Hirshfeld surface of (I), Table 4 ▸, arises from a significant number of methyl-C—H⋯π(phen­yl) inter­actions (Tables 2 ▸ and 3 ▸) and short phenyl-, pyrazolyl-C⋯H(water, meth­yl) contacts (Table 3 ▸). The presence of C—H⋯π inter­actions are viewed as the pair of characteristic wings in Fig. 8 ▸ *e* with the shortest C⋯H contact represented as the pair of peaks at *d*_e_ + *d*_i_ ∼2.7 Å, Table 3 ▸. It is evident from the fingerprint plots delin­eated into C⋯C and C⋯N/N⋯C contacts in Fig. 8 ▸ *f* and *g*, arise from the presence of inter-layer π–π contacts between pyrazolyl and phenyl rings whereas the other short C⋯C contacts summarized in Table 3 ▸ are intra-layer, *i.e*. methyl-C8⋯C22(meth­yl). The small contribution from C⋯O/O⋯C contacts appears to have a negligible effect on the mol­ecular packing.

## Database survey   

There are no direct precedents for the neutral mol­ecule found in (I) in the crystallographic literature. Arguably, the most closely related species is the compound whereby the nitro­gen-bound proton in the pendant five-membered ring has been substituted by a phenyl ring to give (II) – this structure has been reported three times [Bertolasi *et al.*, 1995 ▸ (ZILJIN); Kumar *et al.*, 1995 ▸ (ZILJIN01); Ghandour *et al.*, 2017 ▸ (ZILJIN02)]. Here, owing to the presence of the phenyl ring, there is a significant twist between the five-membered rings as seen in the C_carbon­yl_—C—C—N_external ring_ torsion angle of 57.1 (3)°. In two other derivatives, a similar situation pertains. In the derivative where the original phenyl ring of the neutral mol­ecule in (I) is substituted with a benzene­sulfonamide group (EXIJEB; Asiri *et al.*, 2011 ▸), the equivalent torsion angle is 132.9 (2)°. Finally, when both phenyl groups of (II) are substituted with 4-chloro­benzene rings (KUZPIF; Rabnawaz *et al.*, 2010 ▸), C_carbon­yl_—C—C—N_external ring_ torsion angles of −57 (1) and 56 (1)° are found for the two crystallographically independent mol­ecules comprising the asymmetric unit.

## Synthesis and crystallization   

4-Acetoacetyl-3-methyl-1-phenyl-2-pyrazolin-5-one 1 (2.5 g, 10 mmol) and hydrazin hydrazine (1 ml) were refluxed in a mixture of ethanol (50 ml) and acetic acid (50 ml) for 2 h. The reaction mixture was allowed to stand at room temperature. The precipitate was filtered and recrystallized from ethanol solution as fine needles, M.p. 409–410 K. Yield: 70%.

## Refinement details   

Crystal data, data collection and structure refinement details are summarized in Table 6 ▸. The carbon-bound H atoms were placed in calculated positions (C—H = 0.95–0.98 Å) and were included in the refinement in the riding-model approximation, with *U*_iso_(H) set to 1.2–1.5*U*_eq_(C). The O- and N-bound H atoms were refined with distance restraints of 0.84±0.01 and 0.86±0.01 Å, respectively, and with *U*_iso_(H) = 1.5*U*_eq_(O) and 1.2*U*_eq_(N).

## Supplementary Material

Crystal structure: contains datablock(s) I, global. DOI: 10.1107/S2056989019004389/hb7813sup1.cif

Structure factors: contains datablock(s) I. DOI: 10.1107/S2056989019004389/hb7813Isup2.hkl

Supporting information file. DOI: 10.1107/S2056989019004389/hb7813Isup3.cml

CCDC reference: 1906841

Additional supporting information:  crystallographic information; 3D view; checkCIF report

## Figures and Tables

**Table 1:** Selected geometric parameters (Å, °) for (I)

Atoms	Parameter	Atoms	Parameter
C1—O1	1.266 (2)	C15—O2	1.263 (2)
N1—N2	1.376 (2)	N5—N6	1.401 (2)
C1—N1	1.389 (2)	C15—N5	1.396 (2)
C2—N2	1.338 (2)	C17—N6	1.329 (2)
C1—C3	1.429 (2)	C15—C17	1.416 (2)
C2—C3	1.391 (2)	C16—C17	1.405 (2)
N3—N4	1.364 (2)	N7—N8	1.349 (2)
C5—N3	1.346 (2)	C19—N7	1.349 (2)
C7—N4	1.338 (2)	C21—N8	1.337 (2)
C5—C6	1.414 (2)	C19—C20	1.404 (2)
C6—C7	1.375 (2)	C20—C21	1.389 (2)
N2—N1—C1	109.07 (13)	C15—N5—N6	111.51 (13)
N2—N1—C9	121.16 (14)	N6—N5—C23	119.25 (14)
C1—N1—C9	129.62 (14)	C15—N5—C23	128.95 (15)
N1—N2—C2	108.78 (14)	N5—N6—C16	105.37 (13)
N4—N3—C5	103.94 (14)	N8—N7—C19	110.58 (14)
N3—N4—C7	113.60 (14)	N7—N8—C21	108.36 (14)

**Table 2:** Hydrogen-bond geometry (Å, °) *Cg*1 is the centroid of the (C23–C28) ring.

*D*—H⋯*A*	*D*—H	H⋯*A*	*D*⋯*A*	*D*—H⋯*A*
N2—H2*N*⋯O1*W* ^i^	0.90 (1)	1.79 (1)	2.673 (2)	168 (2)
N4—H4*N*⋯O2	0.88 (1)	1.90 (1)	2.764 (2)	168 (2)
N7—H7*N*⋯N3	0.89 (1)	2.28 (2)	2.970 (2)	134 (1)
N8—H8*N*⋯O1	0.90 (1)	1.79 (1)	2.664 (2)	166 (2)
O1*W*—H1*W*⋯O1^ii^	0.85 (2)	1.92 (2)	2.7641 (17)	172 (2)
O1*W*—H2*W*⋯N6	0.86 (2)	1.94 (2)	2.7979 (19)	178 (2)
C8—H8*A*⋯*Cg*1^iii^	0.98	2.71	3.492 (2)	137
C8—H8*B*⋯*Cg*1^iv^	0.98	2.89	3.755 (2)	148

Symmetry codes: (i) 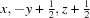; (ii) 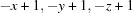; (iii) 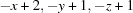; (iv) 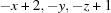.

**Table 3:** Summary of short inter­atomic contacts (Å) in (I)

Contact	Distance	Symmetry operation
H4*N*⋯H7*N*	2.05	*x*, *y*, *z*
H13⋯H18*A*	2.28	*x*, 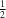 − *y*, 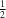 + *z*
H14⋯H18*A*	2.05	*x*, 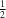 − *y*, 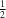 + *z*
C1⋯H1*W*	2.60	1 − *x*, 1 − *y*, 1 − *z*
C10⋯H1*W*	2.81	1 − *x*, 1 − *y*, 1 − *z*
C14⋯H18*A*	2.75	*x*, 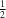 − *y*, 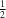 + *z*
C28⋯H8*B*	2.75	2 − *x*, − *y*, 1 − *z*
N2⋯C14	3.238 (2)	1 − *x*, − 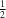 + *y*, 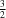 − *z*
C2⋯C13	3.382 (3)	1 − *x*, − 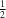 + *y*, 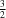 − *z*
C8⋯C22	3.317 (2)	1 + *x*, *y*, *z*
C15⋯C22	3.352 (3)	1 − *x*, 1 − *y*, 1 − *z*
C17⋯C21	3.391 (3)	1 − *x*, 1 − *y*, 1 − *z*

**Table 4:** Percentage contributions of inter­atomic contacts to the Hirshfeld surface for (I)

Contact		Percentage contribution	
	neutral tautomer	zwitterion	overall
H⋯H	49.8	52.4	52.4
O⋯H/H⋯O	11.1	9.4	8.8
N⋯H/H⋯N	8.2	14.0	9.1
C⋯H/H⋯C	21.5	15.1	19.9
C⋯C	5.2	5.9	5.9
C⋯N/N⋯C	4.9	3.1	3.7
C⋯O/O⋯C	0.2	0.1	0.2

**Table 5:** Geometric data (Å) for additional π–π inter­actions in the crystal of (I)

First ring	Second ring	Separation	Symmetry operation
*Cg*(N1,N2,C1–C3)	*Cg*(C9–C14)	3.5810 (9)	1 − *x*, 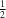 + *y*, 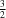 − *z*
*Cg*(N5,N6,C15–C17)	*Cg*(N7,N8,C19–C21)	3.8064 (9)	1 − *x*, 1 − *y*, 1 − *z*
*Cg* (N7,N8,C19–C21)	*Cg*(N7,N8,C19–C21)	3.6886 (10)	1 − *x*, − *y*, 1 − *z*

**Table 6:** Experimental details

Crystal data	
Chemical formula	2C_14_H_14_N_4_O·H_2_O
*M*_r_	526.60
Crystal system, space group	Monoclinic, *P*2_1_/*c*
Temperature (K)	100
*a*, *b*, *c* (Å)	11.7007 (6), 7.0419 (3), 31.2567 (17)
β (°)	98.379 (5)
*V* (Å^3^)	2547.9 (2)
*Z*	4
Radiation type	Mo *K*α
μ (mm^−1^)	0.09
Crystal size (mm)	0.35 × 0.35 × 0.05

Data collection	
Diffractometer	Agilent SuperNova Dual diffractometer with an Atlas detector
Absorption correction	Multi-scan (*CrysAlis PRO*; Agilent, 2011 ▸)
*T*_min_, *T*_max_	0.968, 0.995
No. of measured, independent and observed [*I* > 2σ(*I*)] reflections	10823, 5838, 4411
*R*_int_	0.031
(sin θ/λ)_max_ (Å^−1^)	0.651

Refinement	
*R*[*F* ^2^ > 2σ(*F* ^2^)], *wR*(*F* ^2^), *S*	0.052, 0.137, 1.06
No. of reflections	5838
No. of parameters	374
No. of restraints	6
H-atom treatment	H atoms treated by a mixture of independent and constrained refinement
Δρ_max_, Δρ_min_ (e Å^−3^)	0.27, −0.41

Computer programs: *CrysAlis PRO* (Agilent, 2011 ▸), *SHELXS97* (Sheldrick, 2015*a* ▸), *SHELXL2018* (Sheldrick, 2015*b* ▸), *ORTEP-3 for Windows* (Farrugia, 2012 ▸), *QMol*Gans & Shalloway (2001 ▸), *DIAMOND* (Brandenburg, 2006 ▸) and *publCIF* (Westrip, 2010 ▸).

## supplementary crystallographic information

### Crystal data

**Table:**

2C_14_H_14_N_4_O·H_2_O	*F*(000) = 1112
*M**_r_* = 526.60	*D*_x_ = 1.373 Mg m^−^^3^
Monoclinic, *P*2_1_/*c*	Mo *K*α radiation, λ = 0.71073 Å
*a* = 11.7007 (6) Å	Cell parameters from 3750 reflections
*b* = 7.0419 (3) Å	θ = 2.3–27.5°
*c* = 31.2567 (17) Å	µ = 0.09 mm^−^^1^
β = 98.379 (5)°	*T* = 100 K
*V* = 2547.9 (2) Å^3^	Plate, colourless
*Z* = 4	0.35 × 0.35 × 0.05 mm

### Data collection

**Table:**

Agilent SuperNova Dual diffractometer with an Atlas detector	5838 independent reflections
Radiation source: SuperNova (Mo) X-ray Source	4411 reflections with *I* > 2σ(*I*)
Mirror monochromator	*R*_int_ = 0.031
Detector resolution: 10.4041 pixels mm^-1^	θ_max_ = 27.6°, θ_min_ = 2.4°
ω scan	*h* = −15→9
Absorption correction: multi-scan (CrysAlis PRO; Agilent, 2011)	*k* = −5→9
*T*_min_ = 0.968, *T*_max_ = 0.995	*l* = −40→39
10823 measured reflections	

### Refinement

**Table:**

Refinement on *F*^2^	Primary atom site location: structure-invariant direct methods
Least-squares matrix: full	Hydrogen site location: mixed
*R*[*F*^2^ > 2σ(*F*^2^)] = 0.052	H atoms treated by a mixture of independent and constrained refinement
*wR*(*F*^2^) = 0.137	*w* = 1/[σ^2^(*F*_o_^2^) + (0.0593*P*)^2^ + 0.7451*P*] where *P* = (*F*_o_^2^ + 2*F*_c_^2^)/3
*S* = 1.06	(Δ/σ)_max_ = 0.001
5838 reflections	Δρ_max_ = 0.27 e Å^−^^3^
374 parameters	Δρ_min_ = −0.40 e Å^−^^3^
6 restraints	

### Special details

**Table:**

Geometry. All esds (except the esd in the dihedral angle between two l.s. planes) are estimated using the full covariance matrix. The cell esds are taken into account individually in the estimation of esds in distances, angles and torsion angles; correlations between esds in cell parameters are only used when they are defined by crystal symmetry. An approximate (isotropic) treatment of cell esds is used for estimating esds involving l.s. planes.

### Fractional atomic coordinates and isotropic or equivalent isotropic displacement parameters (Å^2^)

**Table:**

	*x*	*y*	*z*	*U*_iso_*/*U*_eq_	
O1	0.50164 (10)	0.12787 (17)	0.64929 (4)	0.0181 (3)	
N1	0.53780 (11)	0.0341 (2)	0.72110 (5)	0.0153 (3)	
N2	0.63356 (12)	0.0066 (2)	0.75165 (5)	0.0160 (3)	
N3	0.73563 (12)	0.1334 (2)	0.61575 (5)	0.0210 (3)	
H2N	0.6292 (16)	−0.025 (3)	0.7792 (4)	0.025*	
N4	0.83286 (12)	0.1666 (2)	0.59759 (5)	0.0208 (3)	
H4N	0.8238 (17)	0.188 (3)	0.5695 (3)	0.025*	
C1	0.57339 (14)	0.0824 (2)	0.68201 (5)	0.0148 (3)	
C2	0.72834 (14)	0.0263 (2)	0.73272 (6)	0.0159 (4)	
C3	0.69669 (14)	0.0740 (2)	0.68942 (5)	0.0150 (3)	
C4	0.84351 (14)	−0.0047 (3)	0.75912 (6)	0.0194 (4)	
H4A	0.833118	−0.055901	0.787460	0.029*	
H4B	0.885122	0.116239	0.762975	0.029*	
H4C	0.888010	−0.095029	0.744297	0.029*	
C5	0.77502 (14)	0.1141 (2)	0.65819 (6)	0.0150 (3)	
C6	0.89639 (14)	0.1367 (2)	0.66601 (6)	0.0172 (4)	
H6	0.944899	0.130460	0.693134	0.021*	
C7	0.92969 (14)	0.1697 (2)	0.62624 (6)	0.0171 (4)	
C8	1.04501 (14)	0.1965 (3)	0.61202 (6)	0.0212 (4)	
H8A	1.039270	0.293826	0.589397	0.032*	
H8B	1.070308	0.076438	0.600611	0.032*	
H8C	1.101164	0.236834	0.636718	0.032*	
C9	0.42443 (14)	0.0244 (2)	0.73226 (6)	0.0151 (3)	
C10	0.33184 (14)	−0.0258 (2)	0.70096 (6)	0.0177 (4)	
H10	0.344090	−0.057124	0.672419	0.021*	
C11	0.22132 (14)	−0.0291 (2)	0.71232 (6)	0.0196 (4)	
H11	0.157180	−0.057578	0.690979	0.024*	
C12	0.20340 (14)	0.0087 (2)	0.75446 (6)	0.0196 (4)	
H12	0.127628	0.003740	0.761951	0.024*	
C13	0.29632 (14)	0.0537 (3)	0.78551 (6)	0.0193 (4)	
H13	0.284374	0.077360	0.814448	0.023*	
C14	0.40700 (14)	0.0643 (2)	0.77450 (6)	0.0178 (4)	
H14	0.470414	0.098477	0.795649	0.021*	
O2	0.77522 (10)	0.25890 (19)	0.51120 (4)	0.0248 (3)	
N5	0.74784 (12)	0.3174 (2)	0.43598 (5)	0.0155 (3)	
N6	0.65481 (12)	0.3330 (2)	0.40247 (5)	0.0173 (3)	
N7	0.56152 (12)	0.2282 (2)	0.53983 (5)	0.0178 (3)	
H7N	0.6363 (9)	0.222 (3)	0.5506 (6)	0.021*	
N8	0.47846 (12)	0.1938 (2)	0.56454 (5)	0.0177 (3)	
H8N	0.4983 (16)	0.166 (3)	0.5926 (3)	0.021*	
C15	0.71023 (14)	0.2837 (2)	0.47567 (6)	0.0165 (4)	
C16	0.56071 (14)	0.3123 (2)	0.42123 (6)	0.0163 (4)	
C17	0.58821 (14)	0.2820 (2)	0.46598 (5)	0.0153 (3)	
C18	0.44402 (15)	0.3236 (3)	0.39471 (6)	0.0237 (4)	
H18A	0.452353	0.350197	0.364543	0.036*	
H18B	0.399461	0.425608	0.405806	0.036*	
H18C	0.403635	0.202539	0.396371	0.036*	
C19	0.51492 (14)	0.2495 (2)	0.49802 (5)	0.0151 (3)	
C20	0.39512 (14)	0.2301 (2)	0.49695 (6)	0.0169 (4)	
H20	0.337894	0.239186	0.472140	0.020*	
C21	0.37622 (14)	0.1949 (2)	0.53908 (6)	0.0166 (4)	
C22	0.26704 (15)	0.1657 (3)	0.55739 (6)	0.0220 (4)	
H22A	0.282458	0.086869	0.583433	0.033*	
H22B	0.210356	0.102077	0.535950	0.033*	
H22C	0.236420	0.288951	0.564862	0.033*	
C23	0.86164 (14)	0.3190 (2)	0.42595 (6)	0.0158 (4)	
C24	0.95656 (15)	0.3283 (3)	0.45857 (6)	0.0241 (4)	
H24	0.945472	0.331769	0.488071	0.029*	
C25	1.06731 (16)	0.3324 (3)	0.44760 (7)	0.0287 (5)	
H25	1.131685	0.340211	0.469881	0.034*	
C26	1.08599 (15)	0.3254 (3)	0.40489 (6)	0.0248 (4)	
H26	1.162274	0.326228	0.397846	0.030*	
C27	0.99162 (15)	0.3172 (3)	0.37267 (6)	0.0207 (4)	
H27	1.003385	0.313770	0.343246	0.025*	
C28	0.87980 (14)	0.3139 (2)	0.38275 (6)	0.0180 (4)	
H28	0.815760	0.308261	0.360297	0.022*	
O1W	0.65340 (10)	0.59378 (18)	0.33486 (4)	0.0195 (3)	
H1W	0.6112 (15)	0.686 (2)	0.3408 (7)	0.029*	
H2W	0.6537 (17)	0.512 (2)	0.3552 (5)	0.029*	

### Atomic displacement parameters (Å^2^)

**Table:**

	*U*^11^	*U*^22^	*U*^33^	*U*^12^	*U*^13^	*U*^23^
O1	0.0169 (6)	0.0268 (7)	0.0107 (6)	0.0025 (5)	0.0026 (5)	0.0029 (5)
N1	0.0152 (7)	0.0215 (7)	0.0092 (7)	−0.0003 (6)	0.0020 (5)	0.0006 (6)
N2	0.0162 (7)	0.0228 (7)	0.0090 (7)	−0.0007 (6)	0.0017 (6)	0.0018 (6)
N3	0.0169 (7)	0.0347 (9)	0.0125 (8)	−0.0008 (6)	0.0064 (6)	0.0028 (7)
N4	0.0173 (7)	0.0344 (9)	0.0117 (8)	0.0005 (6)	0.0052 (6)	0.0054 (7)
C1	0.0185 (8)	0.0150 (8)	0.0116 (8)	−0.0001 (6)	0.0042 (7)	−0.0001 (7)
C2	0.0183 (8)	0.0167 (8)	0.0136 (9)	−0.0010 (6)	0.0047 (7)	0.0004 (7)
C3	0.0164 (8)	0.0174 (8)	0.0114 (8)	0.0005 (6)	0.0031 (6)	0.0009 (7)
C4	0.0182 (8)	0.0248 (9)	0.0150 (9)	−0.0011 (7)	0.0016 (7)	0.0021 (7)
C5	0.0168 (8)	0.0161 (8)	0.0124 (8)	−0.0002 (6)	0.0035 (7)	−0.0008 (7)
C6	0.0158 (8)	0.0238 (9)	0.0122 (9)	0.0001 (7)	0.0023 (7)	0.0012 (7)
C7	0.0180 (8)	0.0185 (8)	0.0158 (9)	0.0023 (7)	0.0056 (7)	0.0025 (7)
C8	0.0186 (9)	0.0255 (9)	0.0215 (10)	0.0017 (7)	0.0092 (7)	0.0046 (8)
C9	0.0162 (8)	0.0160 (8)	0.0141 (9)	0.0008 (6)	0.0054 (7)	0.0038 (7)
C10	0.0213 (8)	0.0199 (8)	0.0124 (9)	−0.0004 (7)	0.0038 (7)	0.0014 (7)
C11	0.0188 (8)	0.0212 (9)	0.0180 (9)	−0.0016 (7)	0.0000 (7)	0.0011 (7)
C12	0.0170 (8)	0.0224 (9)	0.0206 (10)	−0.0006 (7)	0.0068 (7)	0.0012 (8)
C13	0.0211 (9)	0.0255 (9)	0.0126 (9)	0.0021 (7)	0.0065 (7)	0.0005 (7)
C14	0.0177 (8)	0.0234 (9)	0.0123 (9)	0.0007 (7)	0.0023 (7)	0.0011 (7)
O2	0.0178 (6)	0.0448 (8)	0.0114 (6)	−0.0039 (6)	0.0005 (5)	0.0048 (6)
N5	0.0149 (7)	0.0214 (7)	0.0103 (7)	0.0001 (6)	0.0025 (5)	0.0021 (6)
N6	0.0153 (7)	0.0249 (8)	0.0111 (7)	0.0002 (6)	0.0003 (6)	0.0011 (6)
N7	0.0153 (7)	0.0268 (8)	0.0118 (7)	−0.0001 (6)	0.0038 (6)	0.0005 (6)
N8	0.0192 (7)	0.0247 (8)	0.0101 (7)	−0.0011 (6)	0.0050 (6)	0.0012 (6)
C15	0.0185 (8)	0.0197 (8)	0.0116 (8)	−0.0021 (7)	0.0031 (7)	−0.0008 (7)
C16	0.0176 (8)	0.0197 (8)	0.0122 (9)	0.0002 (7)	0.0043 (7)	0.0011 (7)
C17	0.0167 (8)	0.0182 (8)	0.0115 (8)	−0.0014 (6)	0.0036 (6)	−0.0008 (7)
C18	0.0165 (8)	0.0415 (11)	0.0132 (9)	−0.0004 (8)	0.0023 (7)	0.0054 (8)
C19	0.0182 (8)	0.0166 (8)	0.0109 (8)	−0.0002 (6)	0.0032 (6)	−0.0012 (7)
C20	0.0161 (8)	0.0222 (9)	0.0124 (9)	−0.0011 (7)	0.0027 (6)	−0.0001 (7)
C21	0.0181 (8)	0.0169 (8)	0.0155 (9)	−0.0002 (6)	0.0046 (7)	0.0006 (7)
C22	0.0220 (9)	0.0267 (9)	0.0190 (10)	−0.0002 (7)	0.0094 (7)	0.0006 (8)
C23	0.0156 (8)	0.0176 (8)	0.0151 (9)	0.0000 (6)	0.0053 (7)	0.0016 (7)
C24	0.0199 (9)	0.0377 (11)	0.0148 (9)	−0.0012 (8)	0.0033 (7)	0.0013 (8)
C25	0.0189 (9)	0.0462 (12)	0.0206 (10)	−0.0001 (8)	0.0014 (8)	0.0044 (9)
C26	0.0174 (9)	0.0330 (10)	0.0257 (11)	0.0013 (8)	0.0085 (8)	0.0033 (8)
C27	0.0214 (9)	0.0252 (9)	0.0172 (9)	0.0017 (7)	0.0090 (7)	0.0000 (7)
C28	0.0180 (8)	0.0224 (9)	0.0136 (9)	0.0002 (7)	0.0027 (7)	0.0000 (7)
O1W	0.0239 (7)	0.0224 (6)	0.0131 (7)	0.0050 (5)	0.0058 (5)	0.0022 (5)

### Geometric parameters (Å, º)

**Table:**

O1—C1	1.266 (2)	N5—C15	1.396 (2)
N1—N2	1.3759 (19)	N5—N6	1.4006 (19)
N1—C1	1.389 (2)	N5—C23	1.412 (2)
N1—C9	1.422 (2)	N6—C16	1.329 (2)
N2—C2	1.338 (2)	N7—N8	1.3486 (19)
N2—H2N	0.898 (9)	N7—C19	1.349 (2)
N3—C5	1.346 (2)	N7—H7N	0.892 (9)
N3—N4	1.3636 (19)	N8—C21	1.337 (2)
N4—C7	1.338 (2)	N8—H8N	0.894 (9)
N4—H4N	0.882 (9)	C15—C17	1.416 (2)
C1—C3	1.429 (2)	C16—C17	1.405 (2)
C2—C3	1.391 (2)	C16—C18	1.493 (2)
C2—C4	1.490 (2)	C17—C19	1.429 (2)
C3—C5	1.461 (2)	C18—H18A	0.9800
C4—H4A	0.9800	C18—H18B	0.9800
C4—H4B	0.9800	C18—H18C	0.9800
C4—H4C	0.9800	C19—C20	1.404 (2)
C5—C6	1.414 (2)	C20—C21	1.389 (2)
C6—C7	1.375 (2)	C20—H20	0.9500
C6—H6	0.9500	C21—C22	1.488 (2)
C7—C8	1.493 (2)	C22—H22A	0.9800
C8—H8A	0.9800	C22—H22B	0.9800
C8—H8B	0.9800	C22—H22C	0.9800
C8—H8C	0.9800	C23—C24	1.396 (2)
C9—C14	1.394 (2)	C23—C28	1.397 (2)
C9—C10	1.395 (2)	C24—C25	1.388 (2)
C10—C11	1.390 (2)	C24—H24	0.9500
C10—H10	0.9500	C25—C26	1.385 (3)
C11—C12	1.389 (2)	C25—H25	0.9500
C11—H11	0.9500	C26—C27	1.383 (3)
C12—C13	1.384 (2)	C26—H26	0.9500
C12—H12	0.9500	C27—C28	1.390 (2)
C13—C14	1.390 (2)	C27—H27	0.9500
C13—H13	0.9500	C28—H28	0.9500
C14—H14	0.9500	O1W—H1W	0.851 (9)
O2—C15	1.263 (2)	O1W—H2W	0.858 (9)
			
N2—N1—C1	109.07 (13)	C15—N5—C23	128.95 (15)
N2—N1—C9	121.16 (14)	N6—N5—C23	119.25 (14)
C1—N1—C9	129.62 (14)	C16—N6—N5	105.37 (13)
C2—N2—N1	108.78 (14)	N8—N7—C19	110.58 (14)
C2—N2—H2N	128.0 (12)	N8—N7—H7N	121.6 (13)
N1—N2—H2N	123.1 (12)	C19—N7—H7N	127.4 (13)
C5—N3—N4	103.94 (14)	C21—N8—N7	108.36 (14)
C7—N4—N3	113.60 (14)	C21—N8—H8N	131.8 (13)
C7—N4—H4N	129.1 (13)	N7—N8—H8N	119.6 (13)
N3—N4—H4N	117.2 (13)	O2—C15—N5	125.28 (15)
O1—C1—N1	121.59 (14)	O2—C15—C17	130.23 (16)
O1—C1—C3	132.57 (15)	N5—C15—C17	104.49 (14)
N1—C1—C3	105.80 (14)	N6—C16—C17	111.82 (15)
N2—C2—C3	109.58 (15)	N6—C16—C18	119.93 (15)
N2—C2—C4	118.72 (15)	C17—C16—C18	128.26 (15)
C3—C2—C4	131.70 (15)	C16—C17—C15	106.80 (14)
C2—C3—C1	106.69 (14)	C16—C17—C19	130.42 (16)
C2—C3—C5	126.36 (15)	C15—C17—C19	122.76 (15)
C1—C3—C5	126.92 (15)	C16—C18—H18A	109.5
C2—C4—H4A	109.5	C16—C18—H18B	109.5
C2—C4—H4B	109.5	H18A—C18—H18B	109.5
H4A—C4—H4B	109.5	C16—C18—H18C	109.5
C2—C4—H4C	109.5	H18A—C18—H18C	109.5
H4A—C4—H4C	109.5	H18B—C18—H18C	109.5
H4B—C4—H4C	109.5	N7—C19—C20	105.87 (15)
N3—C5—C6	110.48 (15)	N7—C19—C17	119.85 (15)
N3—C5—C3	121.32 (15)	C20—C19—C17	134.28 (16)
C6—C5—C3	128.19 (16)	C21—C20—C19	106.93 (15)
C7—C6—C5	105.80 (15)	C21—C20—H20	126.5
C7—C6—H6	127.1	C19—C20—H20	126.5
C5—C6—H6	127.1	N8—C21—C20	108.26 (14)
N4—C7—C6	106.17 (15)	N8—C21—C22	120.92 (16)
N4—C7—C8	121.10 (16)	C20—C21—C22	130.81 (16)
C6—C7—C8	132.67 (16)	C21—C22—H22A	109.5
C7—C8—H8A	109.5	C21—C22—H22B	109.5
C7—C8—H8B	109.5	H22A—C22—H22B	109.5
H8A—C8—H8B	109.5	C21—C22—H22C	109.5
C7—C8—H8C	109.5	H22A—C22—H22C	109.5
H8A—C8—H8C	109.5	H22B—C22—H22C	109.5
H8B—C8—H8C	109.5	C24—C23—C28	119.36 (15)
C14—C9—C10	120.62 (15)	C24—C23—N5	120.93 (16)
C14—C9—N1	119.57 (15)	C28—C23—N5	119.70 (15)
C10—C9—N1	119.81 (15)	C25—C24—C23	119.51 (18)
C11—C10—C9	118.77 (16)	C25—C24—H24	120.2
C11—C10—H10	120.6	C23—C24—H24	120.2
C9—C10—H10	120.6	C26—C25—C24	121.44 (18)
C12—C11—C10	120.89 (16)	C26—C25—H25	119.3
C12—C11—H11	119.6	C24—C25—H25	119.3
C10—C11—H11	119.6	C27—C26—C25	118.82 (16)
C13—C12—C11	119.79 (16)	C27—C26—H26	120.6
C13—C12—H12	120.1	C25—C26—H26	120.6
C11—C12—H12	120.1	C26—C27—C28	120.91 (17)
C12—C13—C14	120.26 (16)	C26—C27—H27	119.5
C12—C13—H13	119.9	C28—C27—H27	119.5
C14—C13—H13	119.9	C27—C28—C23	119.95 (16)
C13—C14—C9	119.61 (16)	C27—C28—H28	120.0
C13—C14—H14	120.2	C23—C28—H28	120.0
C9—C14—H14	120.2	H1W—O1W—H2W	107.0 (19)
C15—N5—N6	111.51 (13)		
			
C1—N1—N2—C2	−3.11 (18)	C15—N5—N6—C16	0.98 (18)
C9—N1—N2—C2	−179.05 (15)	C23—N5—N6—C16	175.29 (15)
C5—N3—N4—C7	−0.2 (2)	C19—N7—N8—C21	−0.87 (19)
N2—N1—C1—O1	−175.10 (14)	N6—N5—C15—O2	178.15 (16)
C9—N1—C1—O1	0.4 (3)	C23—N5—C15—O2	4.5 (3)
N2—N1—C1—C3	2.85 (17)	N6—N5—C15—C17	−1.05 (18)
C9—N1—C1—C3	178.34 (16)	C23—N5—C15—C17	−174.67 (16)
N1—N2—C2—C3	2.06 (19)	N5—N6—C16—C17	−0.50 (19)
N1—N2—C2—C4	−177.61 (14)	N5—N6—C16—C18	179.24 (15)
N2—C2—C3—C1	−0.27 (19)	N6—C16—C17—C15	−0.1 (2)
C4—C2—C3—C1	179.35 (17)	C18—C16—C17—C15	−179.85 (17)
N2—C2—C3—C5	177.71 (15)	N6—C16—C17—C19	−178.60 (17)
C4—C2—C3—C5	−2.7 (3)	C18—C16—C17—C19	1.7 (3)
O1—C1—C3—C2	176.04 (17)	O2—C15—C17—C16	−178.44 (18)
N1—C1—C3—C2	−1.59 (18)	N5—C15—C17—C16	0.70 (18)
O1—C1—C3—C5	−1.9 (3)	O2—C15—C17—C19	0.2 (3)
N1—C1—C3—C5	−179.55 (15)	N5—C15—C17—C19	179.32 (15)
N4—N3—C5—C6	0.35 (19)	N8—N7—C19—C20	1.01 (19)
N4—N3—C5—C3	−179.20 (15)	N8—N7—C19—C17	−178.27 (15)
C2—C3—C5—N3	170.80 (16)	C16—C17—C19—N7	−178.44 (17)
C1—C3—C5—N3	−11.6 (3)	C15—C17—C19—N7	3.3 (3)
C2—C3—C5—C6	−8.7 (3)	C16—C17—C19—C20	2.5 (3)
C1—C3—C5—C6	168.93 (17)	C15—C17—C19—C20	−175.72 (18)
N3—C5—C6—C7	−0.34 (19)	N7—C19—C20—C21	−0.76 (19)
C3—C5—C6—C7	179.16 (16)	C17—C19—C20—C21	178.35 (18)
N3—N4—C7—C6	0.0 (2)	N7—N8—C21—C20	0.35 (19)
N3—N4—C7—C8	177.54 (15)	N7—N8—C21—C22	−178.43 (15)
C5—C6—C7—N4	0.18 (19)	C19—C20—C21—N8	0.26 (19)
C5—C6—C7—C8	−176.92 (18)	C19—C20—C21—C22	178.88 (18)
N2—N1—C9—C14	26.2 (2)	C15—N5—C23—C24	−15.1 (3)
C1—N1—C9—C14	−148.83 (17)	N6—N5—C23—C24	171.73 (15)
N2—N1—C9—C10	−153.49 (15)	C15—N5—C23—C28	165.82 (16)
C1—N1—C9—C10	31.5 (3)	N6—N5—C23—C28	−7.4 (2)
C14—C9—C10—C11	2.1 (3)	C28—C23—C24—C25	0.0 (3)
N1—C9—C10—C11	−178.19 (15)	N5—C23—C24—C25	−179.08 (17)
C9—C10—C11—C12	−2.8 (3)	C23—C24—C25—C26	−0.7 (3)
C10—C11—C12—C13	1.2 (3)	C24—C25—C26—C27	1.0 (3)
C11—C12—C13—C14	1.1 (3)	C25—C26—C27—C28	−0.7 (3)
C12—C13—C14—C9	−1.7 (3)	C26—C27—C28—C23	0.0 (3)
C10—C9—C14—C13	0.1 (3)	C24—C23—C28—C27	0.3 (3)
N1—C9—C14—C13	−179.61 (16)	N5—C23—C28—C27	179.44 (16)

### Hydrogen-bond geometry (Å, º)

*Cg*1 is the centroid of the (C23–C28) ring.

**Table:**

*D*—H···*A*	*D*—H	H···*A*	*D*···*A*	*D*—H···*A*
N2—H2*N*···O1*W*^i^	0.90 (1)	1.79 (1)	2.673 (2)	168 (2)
N4—H4*N*···O2	0.88 (1)	1.90 (1)	2.764 (2)	168 (2)
N7—H7*N*···N3	0.89 (1)	2.28 (2)	2.970 (2)	134 (1)
N8—H8*N*···O1	0.90 (1)	1.79 (1)	2.664 (2)	166 (2)
O1*W*—H1*W*···O1^ii^	0.85 (2)	1.92 (2)	2.7641 (17)	172 (2)
O1*W*—H2*W*···N6	0.86 (2)	1.94 (2)	2.7979 (19)	178 (2)
C8—H8*A*···*Cg*1^iii^	0.98	2.71	3.492 (2)	137
C8—H8*B*···*Cg*1^iv^	0.98	2.89	3.755 (2)	148

Symmetry codes: (i) *x*, −*y*+1/2, *z*+1/2; (ii) −*x*+1, −*y*+1, −*z*+1; (iii) −*x*+2, −*y*+1, −*z*+1; (iv) −*x*+2, −*y*, −*z*+1.
